# Does a Healthy Weight Body Mass Index at Onset of Idiopathic Intracranial Hypertension Change the Outcomes? A United Kingdom Prospective Cohort Study

**DOI:** 10.1080/01658107.2024.2341775

**Published:** 2024-04-22

**Authors:** Mark Thaller, Victoria Homer, Sally Abbott, Jonathan Hazlehurst, Susan P. Mollan, Alexandra J. Sinclair

**Affiliations:** a Translational Brain Science, Institute of Metabolism and Systems Research, University of Birmingham, Birmingham, UK; b Department of Neurology, University Hospitals Birmingham NHS Foundation Trust, Birmingham, UK; c Centre for Endocrinology, Diabetes and Metabolism, Birmingham Health Partners, Birmingham, UK; d Cancer Research (UK) Clinical Trials Unit, University of Birmingham, Birmingham, UK; e Research Centre for Healthcare and Communities, Institute of Health and Wellbeing, Coventry University, Coventry, UK; f Department of Dietetics, University Hospital Coventry and Warwickshire NHS Trust, Coventry, UK; g Department of Diabetes and Endocrinology, University Hospitals Birmingham NHS Foundation Trust, Birmingham, UK; h Institute of Applied Health Research, University of Birmingham, Birmingham, UK; i Birmingham Neuro-Ophthalmology, University Hospitals Birmingham NHS Foundation Trust, Birmingham, UK

**Keywords:** Pseudotumor cerebri, vision, headache, prognosis, weight, OCT imaging, papilloedema

## Abstract

Idiopathic intracranial hypertension (IIH) predominantly affects reproductive-aged females with obesity. However, the prevalence and impact of a healthy weight body mass index (BMI) at disease presentation is not known. This study aimed to evaluate the visual and headache outcomes stratified by the presenting BMI. This was a longitudinal prospective cohort study (IIH Life) based on a tertiary neuro-ophthalmology IIH service, University Hospitals Birmingham NHS Trust, United Kingdom, recruiting consecutive patients living with IIH between 2012 and 2021. Those with a presenting BMI were included. The outcome measures included visual outcomes of LogMAR visual acuity, Humphrey visual field perimetric mean deviation (PMD), optical coherence tomography (OCT) measurements, and headache outcomes of frequency, severity, and Headache Impact Test-6 score. Three hundred seventy-five people with IIH and a documented baseline BMI. About 3.7% of the entire cohort had a healthy weight BMI at IIH presentation and 15.5% BMI < 30 kg/m^2^. The baseline PMD was worse in patients without obesity; however, OCT papilloedema measures were similar. The presence of obesity was associated with a small but significant greater worsening in visual acuity but slower macular ganglion cell layer loss. There was no impact on PMD or papilloedema prognosis related to baseline obesity. The headache outcomes showed heterogeneity, with worse baseline headache frequency in patients with obesity. No BMI group was associated with worse headache outcomes. Patients with a healthy weight BMI or those without obesity at baseline make up a small proportion of IIH patients. BMI at presentation does not appear to influence long-term visual or headache outcomes.

## Introduction

Idiopathic intracranial hypertension (IIH) predominantly affects reproductive-aged females with obesity.^1,2^ IIH is a disease of raised intracranial pressure, which can lead to chronic headaches, visual loss, and cognitive dysfunction.^3–8^ Atypical IIH is a term given to people who meet the diagnostic criteria for IIH but do not fit the typical phenotype, including males or females living without obesity.^3,9^ Obesity is defined by the World Health Organisation as a body mass index (BMI) ≥30 kg/m^2,10^ which is reduced to 27.5 kg/m^2^ in some ethnic groups due to increased cardiometabolic risk at a lower BMI.^11,12^

The adiposity associated with IIH is centripetal^13,14^ and is correlated with lumbar puncture opening pressure.^13^ A two-fold increase in cardiovascular disease risk has been found in people living with IIH, in comparison to age-, sex- and BMI-matched controls.^2^ Other metabolic complications are associated with IIH, such as diabetes mellitus, gestational diabetes, pre-eclampsia, and arterial hypertension.^2,15,16^ In animal studies, both high-fat diet and IIH-associated inflammatory factor-exposed female rats showed increased CSF secretion.^17^ The adipocytes in IIH appear to be metabolically primed for lipogenesis^18^ with an additional insulin and leptin resistant phenotype in excess of that driven by obesity alone.^18,19^ IIH is also associated with hypertestosteronism in females, independent of obesity, and testosterone increases CSF secretion through the Na^+^/K^+^-ATPase pump in the choroid plexus.^20^ There is also a debate in the literature about whether IIH is caused by problems with CSF secretion or drainage (including cerebral venous stenosis), or a combination of both, and multiple factors are likely to influence both these mechanisms.^21,22^

Currently, the only disease-modifying therapy for IIH is weight loss,^3,23^ since weight loss shown to be associated with disease remission.^24–26^ In the IIH weight trial, those with a BMI ≥35 kg/m^2^ disease remission, defined as normalisation of intracranial pressure (ICP), may require up to 24% weight loss.^27^

The presence of a healthy weight BMI on IIH and associated pathophysiology has previously been studied in a single retrospective study, which found better visual outcomes in those with a healthy weight BMI as compared to those with an increased BMI.^9^ The lack of literature in this area may be because a healthy weight BMI at IIH diagnosis may be uncommon. The metabolic alterations related to obesity have been thought to be the main pathological drivers of IIH given the improvement associated with weight loss, what is not known is that, in the subset of IIH with a healthy weight BMI, there may be a different underlying pathogenesis. Therefore, in a rare condition, despite its rising prevalence, it would be difficult to adequately statistically power a trial to assess differing interventions in this subset.^9,28,29^

This study is the first to prospectively assess the prognosis in IIH in a cohort of people with a healthy weight BMI. The hypothesis is that IIH patients without obesity would have different visual and headache outcomes compared to typically obese IIH counterparts.

## Methods

The methodology^5^ of this prospective observational cohort study has been published and ethically approved by NHS National Research Ethics Committee (14/LO/1208), IIH LIFE study. All the patients included in this study attended a specialist IIH clinic (neuro-ophthalmology and neurology joint clinic) at a single tertiary neuroscience centre (University Hospitals Birmingham NHS Foundation Trust (UHB), United Kingdom). All the patients gave written informed consent to participate in this study. The consecutive data were collected between April 2012 and September 2021, for all visits for each patient.

Eligible patients included those who met either a definite or probable diagnosis for IIH based on the 2013 revised diagnostic criteria for IIH^30^ and had a baseline BMI recorded.

The revised diagnostic criteria required for a definite diagnosis of IIH (adapted from Friedman et al.^31^ include:

- the presence of papilloedema,
- a normal neurological examination (except sixth nerve palsy),
- normal brain parenchyma on neuroimaging (no hydrocephalus, mass, structural lesion, or meningeal enhancement), with exclusion of venous sinus thrombosis,
- normal CSF constituents, and
- an elevated lumbar puncture opening pressure ≥25 cm CSF.^30^

A probable IIH diagnosis could be made in a typical patient, where criteria A to D are met. Patients with secondary causes for raised intracranial pressure or IIH without papilloedema were excluded.^3,31^ Secondary causes excluded were cerebral venous sinus thrombosis, mass lesions, infective causes, and medical contributors such as profound anaemia and regular/recent use of tetracyclines. Appropriate imaging was performed in all patients either after first visit or at their previous hospital.

This study stratified patients according to their BMI status at their first visit (baseline) in the specialist clinic. Patients were initially categorised as having (≥30 kg/m^2^) or not having obesity (<30 kg/m^2^). Then, subsequently by World Health Organisation (WHO) BMI categories,^10^: healthy weight BMI (18.5–24.9 kg/m^2^), overweight (25.0–29.9 kg/m^2^), obesity class 1 (30.0–34.9 kg/m^2^) and obesity class 2, and above (≥35.0 kg/m^2^). As IIH has now been recognised as an obesity related disease by the National Institute of Health and Clinical Excellence in the UK, with regard to criteria for referral to a weight management speciality clinic obesity class 2 and above was used as the highest grouping.^12^ BMI groups were not adjusted by ethnicity.

Visual outcomes were: LogMAR visual acuity (measured using Logarithm of the Minimum Angle of Resolution), Humphrey visual field perimetric mean deviation (PMD) (24–2 SITA standard) and optical coherence tomography (OCT, Heidelberg Spectralis™) measures of average global peripapillary retinal nerve fibre layer thickness (RNFL), automatically measured total retinal thickness (TRT) (by the software on the RNFL circle and extracted as TRT) and macular ganglion cell layer (GCL) volume (1, 2.22, 3.45 mm ETDRS plot with the volume measurement as given by the software which would include all nine sectors, by the pre-set macular volume and/or posterior pole methods). To ensure accuracy of segmentation of the retinal layers in moderate-to-severe papilloedema, manual segmentation of RNFL and TRT was performed in peripapillary scans and in cross-sectional slices of optic disc scans for the basement membrane (BM) and inner limiting layer (ILM), where appropriate. During the course of the study, the scanning protocol for GCL changed; however, excellent correlation has previously been published between the macular volume and posterior pole measures (Pearson correlation 0.98).^5^

Headache outcomes were clinically assessed using the following indices: monthly headache days (days/month); monthly migraine-like headache days (days/month); headache severity (0–10 numerical rating scale, where 0 is no pain and 10 equates to the most severe); and headache disability using the Headache Impact Test-6 (HIT-6) score (score is between 36 and 78).

The methods for data collection and statistical analysis have previously been reported.^5^ The statistical analysis was performed using R v4.1.0.^32^ Continuous variables were reported as mean (standard deviation (SD)) and categorical variables as number (percentage). Lme4^33^ used for regression modelling with continuous form of the dependent variables assumed and independent modelling. Statistical significance is indicated by 95% confidence intervals and is met when the intervals do not cross zero or overlap. Adjustments were made for multiple testing. LOESS (locally weighted scatterplot smoothing) graphs were created prior to regression analysis to ascertain the variables’ relationships and any trends.

The study was guided by patient and public involvement of IIHUK, a national patient charity (Registered Charity in England and Wales no 1,143,522 & Scotland SCO43294) that supports carers and patients living with IIH. They provided advice and guidance for the development of the IIH Life questionnaire.

## Results

In this nine-year longitudinal cohort study, 375 people with IIH and a documented baseline BMI were prospectively recruited. A healthy weight BMI was uncommon at presentation representing 3.7% of the cohort (14/375). Patients with higher BMI categories were more common with 11.7% (44/375) classed as overweight, 20% (75/375) obesity class 1, and 64.5% (242/375) obesity class 2 and above (Table 1). Therefore, 15.5% (58/375) had a BMI <30 kg/m^2^, compared to 84.5% (317/375) with obesity. Patients with a healthy weight BMI had the lowest diagnostic CSF opening pressure (Table 1).

**Table 1.** Baseline table by baseline BMI.

	All	Baseline BMI (kg/m2)			
		18.5–24.9	25–29.9	30–34.9	35+
N	375	14	44	75	242
Surgical	53	1	2	14	36
Females	370	13	43	75	239
Age (mean (SD), N), years	31.7 (9.8), 375	26.5 (6.6), 14	33.5 (13.5), 44	31.0 (9.5), 75	31.9 (9.2), 242
BMI (mean (SD), N), kg/m^2^	38.8 (9.1), 375	22.7 (1.4), 14	27.1 (1.6), 44	32.0 (1.5), 75	43.9 (6.8), 242
Diagnostic CSF opening pressure (mean (SD), N), cmCSF	35.6 (8.9), 323	34.8 (6.5), 14	36.5 (9.0), 40	36.5 (11.0), 64	35.1 (8.3), 205

Cerebrospinal fluid diversion surgery rates (during the course of follow-up for this study) were lower for those with a healthy weight BMI at presentation (7%) compared to patients with IIH and higher BMI, with 3.5% of overweight, 19% of obesity class 1, and 15% of obesity class 2+ (Table 1). However, this was not statistically significantly different between any group. Similar proportions of patients were treated with ICP lowering medications in all groups: 57% of healthy weight BMI, 61% of overweight, 63% obesity class 1 and 54% obesity class 2 + .

The duration of follow-up in this cohort seen within our specialist service was a median of 17 months (range 1–86, interquartile range 6–37) for those with at least one follow-up visit. This was analogous to the BMI groups: healthy weight BMI (21 (3–33, 6–25) months), overweight (14 (1–86, 7–37) months), obesity class 1 (19 (1–70, 6–40) months), and obesity class 2 and above (17 (1–80, 7–38) months).

### Presence of obesity and visual outcomes

The presence of obesity did not, however, influence baseline visual acuity, papilloedema measured by OCT (RNFL and TRT), or macular ganglion cell layer volume (Table 2). A BMI <30 kg/m^2^ (normal or overweight BMI) at baseline visit was associated with worse baseline Humphrey visual field PMD than patients with obesity (−5.65 dB (95% CI: −7.51, −3.80) versus −3.20 dB (−3.98, −2.41)) (Table 2).

**Table 2.** Baseline estimates and trajectory from regression modelling for obesity BMI cut-off (30 kg/m^2^).

	Baseline estimate (units)	Change per month (units/month)
LogMAR visual acuity, logunits		
BMI <30	0.0146 (95% CI: −0.0498, 0.079)	0.0033 (95% CI: 0.0014, 0.0052)
BMI >30	0.0201 (95% CI: −0.0076, 0.0478)	−0.0007 (95% CI: −0.0015, 0.0002)
Humphrey visual field PMD, dB		
BMI <30	−5.65 (95% CI: −7.51, −3.8)	0.06 (95% CI: 0, 0.13)
BMI >30	−3.2 (95% CI: −3.98, −2.41)	0.05 (95% CI: 0.02, 0.07)
Global peripapillary retinal nerve fibre layer, µm		
BMI <30	139.84 (95% CI: 119.64, 160.03)	−1.98 (95% CI: −3.18, −0.77)
BMI >30	135.98 (95% CI: 128.09, 143.88)	−1.27 (95% CI: −1.7, −0.84)
Global peripapillary total retinal thickness, µm		
BMI <30	383.16 (95% CI: 354.09, 412.24)	−2.98 (95% CI: −4.49, −1.48)
BMI >30	361.83 (95% CI: 350.78, 372.88)	−1.96 (95% CI: −2.44, −1.47)
Macular ganglion cell layer volume, mm^3^		
BMI <30	0.440533 (95% CI: 0.420416, 0.46065)	−0.000921 (95% CI: −0.001407, −0.000436)
BMI >30	0.442757 (95% CI: 0.434659, 0.450856)	−0.000363 (95% CI: −0.000516, −0.000209)
Headache frequency (days/month)		
BMI <30	15.95 (95% CI: 12.02, 19.87)	−0.12 (95% CI: −0.32, 0.08)
BMI >30	20.32 (95% CI: 18.63, 22)	−0.17 (95% CI: −0.26, −0.07)
Migraine-like headache frequency (days/month)		
BMI <30	8.41 (95% CI: 5.06, 11.77)	− 0.02 (95% CI: −0.22, 0.18)
BMI >30	9.16 (95% CI: 7.69, 10.63)	−0.1 (95% CI: −0.19, −0.01)
Headache severity (VAS 0–10)		
BMI <30	5.34 (95% CI: 4.32, 6.35)	0.01 (95% CI: −0.06, 0.07)
BMI >30	6.19 (95% CI: 5.75, 6.63)	−0.02 (95% CI: −0.05, 0.01)
Headache Impact Test (HIT-6) (score 36–78)		
BMI <30	61.57 (95% CI: 58.04, 65.09)	−0.1 (95% CI: −0.32, 0.13)
BMI >30	61.27 (95% CI: 59.74, 62.80)	−0.01 (95% CI: −0.11, 0.09)

**Figure 1.**
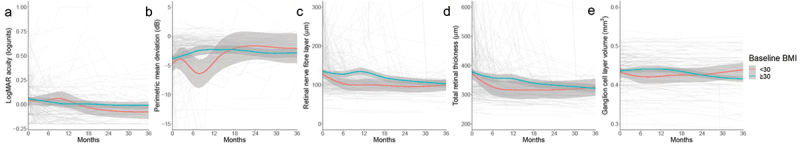
Longitudinal visual data from baseline visit for IIH patients categorised by whether BMI < 30 or ≥30 kg/m^2^, and LOESS smoothers added to show trends across the categories.

Visual outcomes over time, however, were affected differently (Figure 1). Visual acuity had a worse trajectory in those with a BMI <30 kg/m^2^ compared to patients with obesity at baseline (Table 2, Figure 1). There was a small additional statistically significant worsening (increase) in acuity of 0.004 logunits/month (0.002, 0.0061) in the former group compared to the latter, although this would likely remain clinically imperceptible. The PMD trajectory did not differ between the groups with similar long-term outcomes (Figure 1).

(a) LogMAR visual acuity (logunits)

(b) Perimetric mean deviation measured by Humphrey visual field 24-2 testing (dB)

(c) Retinal nerve fibre layer thickness measured on optical coherence tomography (µm)

(d) Total retinal thickness of optic nerve head measured on optical coherence tomography (µm)

(e) Macular ganglion cell layer volume measured on optical coherence tomography (mm^3^).

Papilloedema OCT trajectories were similar between the groups (Table 2); however, in the initial six months following the baseline visit, patients with a BMI <30 kg/m^2^ had a more rapid additional reduction in RNFL of 10.79 µm/month (−26.51, +4.92) and TRT of 5.90 µm/month (−36.29, +24.48) (Figure 1). This was not statistically significant. The macular ganglion cell layer volume declined slightly quicker in patients without obesity (Table 2), with a statistically significant greater decline of 0.000559 mm^3^/month (−0.001068, −0.00005).

### Presence of obesity and headache outcomes

**Figure 2.**
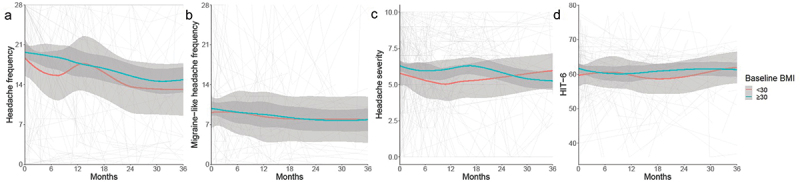
Longitudinal headache data from baseline visit for IIH patients categorised by whether BMI <30 or ≥30 kg/m^2^, and LOESS smoothers added to show trends across the categories.

The headache and migraine-like headache frequencies were high in the IIH cohort at baseline (Table 2, Figure 2). The mean baseline frequency was lower in patients with BMI <30 kg/m^2^ (15.95 days/month (12.02, 19.87) versus 20.32 (18.63, 22.00) with obesity) (Table 2), and this was significantly lower in the non-obesity group by −4.37 days/month (95%CI: −8.64, −0.11)). The migraine-like headache frequency did not significantly differ between patients with or without obesity (9.16 (7.69, 10.63) and 8.41 (5.06, 11.77), respectively) (Table 2, Figure 2). Headache severity was moderate in both groups (Table 2) and did not significantly differ at baseline or in trajectory (Figure 2). Headaches had a substantial impact on the quality of life as measured by the HIT-6, with very similar scores and confidence intervals (baseline scores 61.57 (58.04, 65.09) and 61.27 (59.74, 62.80) for BMI <30 kg/m^2^ and ≥30 kg/m^2^, respectively) (Table 2, Figure 2). The trajectories were not significantly different between <30 kg/m^2^ and ≥30 kg/m^2^ groups for any of the headache outcomes.

(a) Headache frequency (days per month)

(b) Migraine-like headache frequency (days per month)

(c) Headache mean severity of predominant headache (0–10 numerical rating scale)

(d) Headache Impact Test 6 (HIT6) (quality of life measure score 36–78).

### BMI groups and outcomes

**Figure 3.**
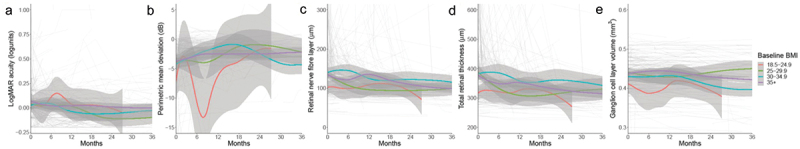
Longitudinal visual data from baseline visit for IIH patients categorised baseline BMI group, and LOESS smoothers added to show trends across the categories.

PMD was significantly worse (more negative) at baseline in those patients with a healthy weight BMI (−9.0 (−12.8, −5.2)) compared to those with obesity class 1 or class 2+ (−2.8 (−4.4, −1.2) and −3.3 (−4.2, −2.4), respectively) (Table 3). It was also worse, but not significantly so, compared to the overweight group (−4.5 (−6.6, −2.4)) (Table 3, Figure 3). OCT TRT papilloedema measurement was significantly worse for overweight patients compared to those with healthy weight BMI, 80.54 µm (12.22, 148.86) higher, but not for either of the obesity groups (Figure 3). Macular GCL at baseline in the healthy weight BMI group was 0.3998 mm^3^ (0.3556, 0.4441) (Table 3) and was significantly higher for both overweight and obesity class 2+ categories compared to healthy weight BMI, 0.0508 (0.0012, 0.1004) and 0.0451 (0, 0.0903), respectively, but not obesity class 1 (Figure 3). When compared to a healthy weight BMI, no other group significantly differed at baseline for visual acuity or OCT RNFL outcomes (Figure 3).

(a) LogMAR visual acuity (logunits)

(b) Perimetric mean deviation measured by Humphrey visual field 24-2 testing (dB)

(c) Retinal nerve fibre layer thickness measured on optical coherence tomography (µm)

(d) Total retinal thickness of optic nerve head measured on optical coherence tomography (µm)

(e) Macular ganglion cell layer volume measured on optical coherence tomography (mm^3^).

**Table 3.** Baseline estimates and trajectory for visual and headache outcomes by baseline BMI from regression modelling.

	Baseline estimate (units)	Change per month (units/month)
LogMAR visual acuity, logunits		
Healthy weight BMI (18.5–24.9 kg/m^2^)	0.0612 (95% CI: −0.0709, 0.1932)	0.0021 (95% CI: −0.002, 0.0062)
Overweight (25.0–29.9 kg/m^2^)	0.0007 (95% CI: −0.0731, 0.0746)	0.0036 (95% CI: 0.0015, 0.0058)
Obesity, class 1 (30.0–34.9 kg/m^2^)	−0.0165 (95% CI: −0.0748, 0.0417)	0.0001 (95% CI: −0.0017, 0.0019)
Obesity, class 2 (35.0+ kg/m^2^)	0.031 (95% CI: −0.0006, 0.0625)	−0.0009 (95% CI: −0.0019, 0)
Humphrey visual field PMD, dB		
Healthy weight BMI (18.5–24.9 kg/m^2^)	−8.9967 (95% CI: −12.7581, −5.2354)	0.0326 (95% CI: −0.1299, 0.1951)
Overweight (25.0–29.9 kg/m^2^)	−4.5161 (95% CI: −6.6363, −2.3958)	0.0685 (95% CI: −0.0009, 0.138)
Obesity, class 1 (30.0–34.9 kg/m^2^)	−2.8149 (95% CI: −4.4375, −1.1923)	0.0253 (95% CI: −0.0243, 0.0749)
Obesity, class 2 (35.0+ kg/m^2^)	−3.321 (95% CI: −4.2083, −2.4336)	0.0564 (95% CI: 0.0243, 0.0885)
Global peripapillary retinal nerve fibre layer, µm		
Healthy weight BMI (18.5–24.9 kg/m^2^)	109.36 (95% CI: 65.85, 152.86)	−1.29 (95% CI: −3.77, 1.18)
Overweight (25.0–29.9 kg/m^2^)	148.22 (95% CI: 125.37, 171.07)	−2.19 (95% CI: −3.56, −0.81)
Obesity, class 1 (30.0–34.9 kg/m^2^)	126.43 (95% CI: 109.82, 143.04)	−1.66 (95% CI: −2.56, −0.77)
Obesity, class 2 (35.0+ kg/m^2^)	138.54 (95% CI: 129.55, 147.53)	−1.16 (95% CI: −1.65, −0.68)
Global peripapillary total retinal thickness, µm		
Healthy weight BMI (18.5–24.9 kg/m^2^)	321.39 (95% CI: 261.78, 381.01)	−2.28 (95% CI: −4.99, 0.43)
Overweight (25.0–29.9 kg/m^2^)	401.94 (95% CI: 368.56, 435.31)	−3.37 (95% CI: −5.17, −1.56)
Obesity, class 1 (30.0–34.9 kg/m^2^)	352.69 (95% CI: 329.5, 375.89)	−2.87 (95% CI: −3.85, −1.88)
Obesity, class 2 (35.0+ kg/m^2^)	363.9 (95% CI: 351.38, 376.42)	−1.68 (95% CI: −2.23, −1.13)
Macular ganglion cell layer volume, mm^3^		
Healthy weight BMI (18.5–24.9 kg/m^2^)	0.3998 (95% CI: 0.3556, 0.4441)	−0.001 (95% CI: −0.0018, −0.0002)
Overweight (25.0–29.9 kg/m^2^)	0.4506 (95% CI: 0.4282, 0.4731)	−0.0009 (95% CI: −0.0015, −0.0003)
Obesity, class 1 (30.0–34.9 kg/m^2^)	0.4352 (95% CI: 0.4183, 0.4521)	−0.0003 (95% CI: −0.0007, 0)
Obesity, class 2 (35.0+ kg/m^2^)	0.445 (95% CI: 0.4358, 0.4541)	−0.0004 (95% CI: −0.0005, −0.0002)
Headache frequency (days/month)		
Healthy weight BMI (18.5–24.9 kg/m^2^)	13.5 (95% CI: 4.72, 22.28)	−0.15 (95% CI: −0.59, 0.28)
Overweight (25.0–29.9 kg/m^2^)	16.6 (95% CI: 12.28, 20.91)	−0.1 (95% CI: −0.34, 0.14)
Obesity, class 1 (30.0–34.9 kg/m^2^)	19.44 (95% CI: 16.34, 22.54)	−0.09 (95% CI: −0.27, 0.08)
Obesity, class 2 (35.0+ kg/m^2^)	20.74 (95% CI: 18.76, 22.72)	−0.2 (95% CI: −0.32, −0.08)
Migraine-like headache frequency (days/month)		
Healthy weight BMI (18.5–24.9 kg/m^2^)	4.34 (95% CI: −2.32, 10.99)	0.16 (95% CI: −0.2, 0.53)
Overweight (25.0–29.9 kg/m^2^)	9.79 (95% CI: 5.97, 13.61)	−0.09 (95% CI: −0.33, 0.15)
Obesity, class 1 (30.0–34.9 kg/m^2^)	6.52 (95% CI: 3.96, 9.07)	0 (95% CI: −0.16, 0.15)
Obesity, class 2 (35.0+ kg/m^2^)	10.46 (95% CI: 8.68, 12.23)	−0.15 (95% CI: −0.26, −0.05)
Headache severity (VAS 0–10)		
Healthy weight BMI (18.5–24.9 kg/m^2^)	5.73 (95% CI: 3.54, 7.93)	−0.09 (95% CI: −0.2, 0.02)
Overweight (25.0–29.9 kg/m^2^)	5.14 (95% CI: 3.99, 6.28)	0.06 (95% CI: −0.02, 0.14)
Obesity, class 1 (30.0–34.9 kg/m^2^)	6.55 (95% CI: 5.67, 7.43)	−0.04 (95% CI: −0.1, 0.01)
Obesity, class 2 (35.0+ kg/m^2^)	6.06 (95% CI: 5.55, 6.57)	−0.01 (95% CI: −0.04, 0.02)
Headache impact test (HIT-6) (score 36–78)		
Healthy weight BMI (18.5–24.9 kg/m^2^)	61.67 (95% CI: 54.4, 68.95)	−0.27 (95% CI: −0.67, 0.13)
Overweight (25.0–29.9 kg/m^2^)	61.33 (95% CI: 57.28, 65.37)	0 (95% CI: −0.27, 0.28)
Obesity, class 1 (30.0–34.9 kg/m^2^)	61.18 (95% CI: 58.19, 64.17)	−0.07 (95% CI: −0.27, 0.14)
Obesity, class 2 (35.0+ kg/m^2^)	61.29 (95% CI: 59.51, 63.08)	0.01 (95% CI: −0.1, 0.13)

Trajectories for all visual outcomes did not differ compared to healthy weight BMI at baseline group (Table 3, Figure 3).

**Figure 4.**
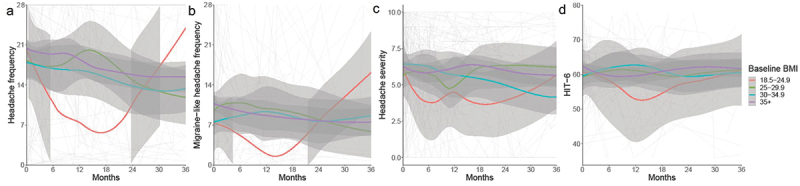
Longitudinal headache data from baseline visit for IIH patients categorised by baseline BMI group, and LOESS smoothers added to show trends across the categories.

Higher headache frequency was reported on average in those with higher baseline BMIs (Table 3, Figure 4). There was no group significantly different from healthy weight BMI at baseline (Figure 4) due to wide confidence intervals. Similarly, migraine-like headache frequency, headache severity, and HIT6 did not differ by BMI group at baseline (Figure 4b–d).

(a) Headache frequency (days per month)

(b) Migraine-like headache frequency (days per month)

(c) Headache mean severity of predominant headache (0–10 numerical rating scale)

(d) Headache Impact Test 6 (HIT6) (quality of life measure score 36–78).

Trajectories for headache outcomes were similar for all outcomes (Table 3, Figure 4) apart from headache severity in the overweight group which had a worse trajectory by 0.15 units/month (0.02, 0.29) compared to healthy weight BMI patients.

## Discussion

In this prospective longitudinal cohort of people living with IIH, a healthy weight BMI at presentation is uncommon. The visual outcomes are similar between BMI groups; however, the improvement in papilloedema was observed to be more rapid in patients without obesity. The headache frequency was higher in IIH patients with obesity as compared to those without, and over time, the headache outcomes were similar between BMI groups.

The prevalence of a healthy weight BMI in this study is similar to a previous large study of IIH.^9^ In this cohort, those with a healthy weight BMI, including those without obesity, had worse visual field function at baseline, despite similar OCT papilloedema measures. This could potentially reflect a delay in diagnosis due to a lower suspicion of IIH in a person with a healthy weight BMI. The OCT imaging measures have been shown to be a more acute marker of disease activity,^5^ as well as a better correlation to intracranial pressure than clinical symptoms.^34,35^ Thus, the comparable OCT measures, with worse PMD, may indicate a delayed presentation of the disease.^5^ The trend seen in CSF diversion surgery rates may reflect that patients with a higher BMI are more likely to present in fulminant IIH and thus require surgery, although this requires further evaluation in other cohorts as was not statistically significant in this cohort. A statistically significant difference was seen in the visual acuity and macular ganglion cell layer volume trajectories between those with obesity and those without (Table 2). However, the clinical significance is uncertain due to the small magnitude of this variation which is likely not be perceivable by the patient or of clinical significance.

The trajectories of the headache outcomes were not significantly different between <30 kg/m^2^ and ≥30 kg/m^2^. This is consistent in that baseline BMI potentially and has a greater influence on visual outcomes than headache outcomes which may be influenced additionally by more significant factors as previous demonstrated in the literature.^5^

Although IIH predominantly affects reproductive-aged women with obesity,^3,5,36,37^ there are atypical presentations.^9,38^ Defining patients by whether obesity was present at baseline visit is clinically useful for potential management of their metabolic syndrome, although this involves an arbitrary cut off at 30 kg/m^2^ which could miss some patients who could benefit. It is important for clinicians to note that baseline BMI did not impact the trajectory of visual or headache outcomes, while for some this could be reassuring; it is important to actively manage those who do not have obesity.

The only current disease-modifying therapy for the systemic manifestations of IIH is weight loss.^3,5,23,26,27^ Its role in patients with a normal baseline BMI is not yet understood. This would be a useful future analysis as a recent analysis of IIH Life database found that a change in BMI was a prognostic marker for visual outcomes.^5^ In other scenarios such as pregnancy, the role of weight loss is not clear.^16,39^

Although the outcomes based on BMI are similar for people living with IIH, it should not be concluded that the underlying pathogenesis is the same. IIH has been shown to be associated with hypertestosteronism independent of obesity,^20^ with metabolic priming towards lipogenesis.^18,19^ In people with obesity, significant weight loss of 24% body weight is often required for disease remission.^25,27,40^ Potential pathogenesis in lean IIH could be inferred from the phenotypically similar condition of polycystic ovarian syndrome (PCOS). In PCOS, one study reported that 42% of the patients had a healthy weight BMI (defined as BMI 18.5–23 kg/m^2^) with a significantly lower prevalence in lean PCOS of deranged lipid profiles, impaired glucose tolerance test, and insulin resistance.^41^ The treatment for insulin resistance with metformin has been shown to lessen hyperandrogenism in PCOS^42^ and has also been shown to potentially be of benefit in IIH.^43^ A limitation of the IIH life database is that metformin use is not routinely documented, and therefore, no further analysis could be done. In IIH with a normal baseline BMI, there may be a role for dietary modifications and pharmacotherapy to avoid future weight gain as this has been shown to be of benefit in lean PCOS.^44^ Anecdotally, clinicians have recommended weight loss in those with a normal BMI where there was a direct history of weight gain prior to IIH diagnosis, as long as not deemed detrimental to the patient’s health.^3^ There is a clear need for further research into the metabolic profile of IIH in those living without obesity, as potentially, there could be a similar metabolic complication risk profile despite the lower body weight, and this could warrant an alternative management approach.

IIH with a healthy weight BMI, or without obesity, may more closely reflect another condition, spaceflight associated neuro-ocular syndrome (SANS) given the healthy weight BMIs observed in astronauts. SANS occurs during long duration spaceflight and risks include hyperopic refractive error shifts, acute visual loss, and cognitive changes. The OCT changes have been noted in SANS with some similarities to IIH, and an increase in the total retinal thickness of 20 µm is used as the earliest indication of optic disc oedema.^45,46^ Translational benefits for both conditions could be established if the underlying pathophysiology was determined.

The major limitation of this study was the relatively small sample size for healthy weight BMI patients. This was similar to a previous study^9^ and likely reflective of the rarity of atypical IIH in an already rare condition. The baseline BMI was used to categorise patients; however, lead-time bias may have been induced with some initial visits being elsewhere prior to tertiary neuro-ophthalmology referral. To minimise this, we created a surrogate marker of disease duration which was defined as the time from the diagnostic lumbar puncture to the first encounter at baseline visit, adjusted for it in the multivariate regression modelling. It is acknowledged that patient reported symptom onset is not recorded within the IIH Life, and this may predate diagnosis by an uncertain and variable duration. Visual function assessments are challenging for patients to perform with both intra-visit and inter-visit factors that are known to influence reliability.^4,47,48^ Given this was a real-world clinical practice study, follow-up intervals were based on the decisions of the attending clinician at each visit and would be influenced by the person’s clinical factors such as disease state, papilloedema severity, and symptomatology. As a result, individuals whose disease was in remission would be more rapidly discharged or their care transferred back to local hospitals. Hence, missing follow-up data increased over the course of time. In addition, some people were lost to follow-up. Therefore, caution in the over interpretation of the long-term outcomes as seen in the LOESS smoother curve should be applied due to the reducing sample size over the course of the study. The diagnosis of IIH is based on the revised diagnostic criteria.^30^ however, due to referral patterns, the cohort contains those with definite and those with probable diagnoses of IIH. It is often challenging to retrieve the initial diagnostic lumbar puncture opening pressure measurement. This analysis prompted and included many further inquiries to external hospital units for this piece of essential clinical data for the clinical record. The lumbar punctures are invasive and can be challenging.^49,50^ It is therefore important that guideline recommendations are adopted to ensure the accurate recording of the opening pressure and help reduce diagnostic uncertainty.^3,48,51^

## Conclusions

People with a normal BMI make up a minority of people diagnosed with IIH. They appear to have similar visual and headache outcomes to people living with IIH and obesity. While at the baseline visit, people without obesity had worse visual field function but similar papilloedema measures, compared to people living with obesity, and their long-term outcomes were similar. The underlying pathophysiology of those without obesity and in whom IIH develops is yet to be discovered.

## Data Availability

Professor Sinclair takes full responsibility for the data, the analyses and interpretation, and the conduct of the research. She has full access to all the data and has the right to publish any and all data separate and apart from any sponsor. Proposals for data access should be made to the corresponding author. Reasonable scientifically sound proposals, from appropriately qualified research groups, will provide data beginning 12 months and ending 3 years after the publication of this article to researchers whose proposed use of the data is approved by the corresponding author. Requesters will need to sign a data access agreement, which will cover the terms and conditions of the release of data and will include publication requirements, authorship, acknowledgements, and obligations for the responsible use of data.
